# Causes of carcass condemnation in Norwegian aviary housed layers

**DOI:** 10.1186/s13028-023-00680-3

**Published:** 2023-05-22

**Authors:** Páll Gretarsson, Käthe Kittelsen, Randi Oppermann Moe, Ingrid Toftaker

**Affiliations:** 1 Department of Production Animal Clinical Sciences, Faculty of Veterinary Medicine, NMBU–Norwegian University of Life Sciences, Ås, Norway; 2 Animalia–The Norwegian Meat and Poultry Research Centre, Oslo, Norway

**Keywords:** Animal welfare, Carcass rejection, DOA, Laying hen, Meat inspection, Poultry health

## Abstract

**Background:**

Meat inspection data is commonly used to monitor health and welfare in commercial broiler production; however, less used in layers. Slaughterhouse records can provide insight into animal and herd health and identify important health and welfare challenges. To gain knowledge of health issues in commercial aviary housed laying hens, the aim of this repeated cross-sectional study was to describe the occurrence and causes of carcass condemnation, including dead-on-arrivals (DOA), in commercial aviary housed layers in Norway, and to explore seasonal patterns and correlation between DOA and number of carcass condemnations.

**Results:**

Data from January 2018 to December 2020 were collected from one poultry abattoir in Norway. In total, 759,584 layers were slaughtered during this period in 101 slaughter batches from 98 flocks and 56 farms. In total, 33,754 (4.4%) layers were condemned, including the DOA. The most common carcass condemnation causes were (percent of all slaughtered layers): abscess/cellulitis (2.03%), peritonitis (0.38%), DOA (0.22%), emaciation (0.22%), discoloration/smell (0.21%), acute skin lesions (0.21%) and ascites (0.17%). Regression analysis showed an estimated higher prevalence of total carcass condemnation during winter compared to the other seasons.

**Conclusions:**

The three most common condemnation causes found in the present study were: abscess/cellulitis, peritonitis, and DOA. We found a large between-batch variation in causes of condemnation and DOA indicating that prevention might be possible. The results can be used to inform and guide further studies on layer health and welfare.

## Background

The main purpose of meat inspection is to ensure food safety and thus support public health. Additionally, meat inspection data are widely used to monitor health and welfare of slaughtered animals [1, 2]. *Ante*- and *post-mortem* inspection provides insight into animal and herd/flock health and welfare, especially towards the end of the production period. Data from meat inspection is an important tool for monitoring health and welfare in poultry, especially broilers, since their production period, inevitably ending in slaughter, is short and intensive. The proportion of birds that die during transport, termed dead on arrival (DOA) is also recorded at the abattoir and used as a welfare and health indicator of the flock [3]. Due to low consumer demand for laying hen meat, only approximately 10% of the total layer population in Norway is slaughtered [4], whereas remaining flocks are euthanized on farm. As a result, meat inspection data has previously not been used routinely to monitor the layer’s health and welfare in the Norwegian egg industry, and hence, knowledge on layer health as measured by carcass condemnation causes is lacking. The European Food Safety Authority (EFSA) recently published an event report where member states discussed the possibilities of using different animal-based measures at slaughter for assessing laying hen welfare on farm [5]. Information on pathology in DOA layers is scarce. Several studies have reported DOA numbers in layers and identified long travel distance and low external temperatures as two main risk factors [3, 6–9]. Body weight has also been reported as a risk factor, where flocks with lighter weighing hens had higher DOA [3, 10]. Internationally, few studies have reported causes of carcass condemnation in laying hens [10, 11]. A study from Portugal reported peritonitis, septicemia, salpingitis, emaciation and tumors to be common causes of condemnation [10], while a study from the Czech Republic reported emaciation and abscesses to be commonly found in laying hens during meat inspection [11]. At farm, common pathological changes have previously been reported to be related to bacterial infections such as colibacillosis and erysipelas, parasitic infections such as coccidiosis or infection with red mite (*Dermanyssus gallinae*) [12, 13] or related to egg laying, e.g. salpingitis, salpingoperitonitis, egg yolk peritonitis and egg impaction [14–16]. Fatty liver is also a prevalent condition in layers [13, 16]. During meat inspection, pathological conditions causing carcass condemnation can give insight into the layer’s health situation. Other condemnation causes that are more acute, e.g., acute skin lesions and acute fractures may indicate welfare challenges during catching, crating, transportation, and uncrating at the abattoir. Documentation of the major health challenges in aviary housed layers is still scarce, and even though the number of slaughtered layers in Norway is small, the information from meat inspection bears the potential as a useful addition to regular health monitoring in live hens during the production period. Thus, the aim of this study was to describe the occurrence and causes of carcass condemnation, including DOA, in commercial aviary housed layers in Norway based on meat inspection data. Additionally, we wanted to explore seasonal patterns and the correlation between DOA and number of carcass condemnations.

## Methods

### Study sample

Meat inspection data on all slaughtered layers from the period 01.01.2018 to 31.12.2020 was provided from one Norwegian poultry slaughterhouse. The study sample consisted of layers from both aviary housing systems and enriched cages. An inclusion criterium was aviary housing, thus the flocks originating from enriched cage systems were excluded. In the following, we will use the term slaughter batch to denote all layers in the same shipment from a farm to the slaughterhouse. One shipment (i.e., birds from the same flock transported on the same day) could in a few cases need more than one transportation vehicle, depending on the size of the flock. Information on the number of vehicles used per shipment was not obtainable. Each flock could also be split and slaughtered at different dates (1–2 days between), resulting in more than one slaughter batch from these flocks. All the received data were aggregated at slaughter-batch level. The dataset included the number of layers in each slaughter batch, the total number of birds condemned, and 14 different causes of condemnation recorded as a count of cases per slaughter batch. Mean carcass weight was also included for each batch.

**Fig. 1:**
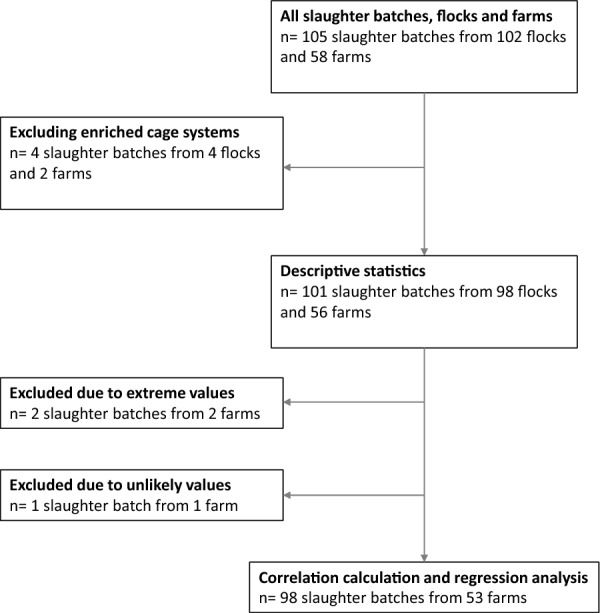
Flowchart of all eligible slaughter batches. Flowchart showing inclusion and exclusion of the study sample and statistical analysis

The 14 different condemnation causes were DOA, abscess/cellulitis, peritonitis, emaciation, discoloration/smell, acute skin lesions, ascites, tumors, fractures, salpingitis, hepatitis, arthritis, damage to the carcass, and fecal contamination. The abscesses recorded with the condemnation cause “abscess/cellulitis” were solely abscesses related to the skin. All condemnations were total carcass condemnations, i.e., partial condemnation of the carcass was not used at the slaughterhouse. Recording of the condemnation cause was performed by veterinarians and veterinary assistants at the abattoir, employed by the official Norwegian Food Safety Authority. Only one condemnation cause was recorded per bird; if a layer had more than one lesion present, the standard approach was to record the most severe lesion as the cause of condemnation. DOA birds where not necropsied/examined. A flow chart describing all eligible slaughter batches, the final study sample as well as inclusions and exclusions for statistical analysis is shown in Fig. 1. Ethical review and approval were waived for this study since it did not involve any interventions. An approval by an ethics committee was therefore not required according to Norwegian legislation [17].

### Data management and statistical analysis

All observations were received as Excel spreadsheets [18]. The data was later transferred to StataSE 16 [19] for cleaning and statistical analysis. Proportions of the different condemnation causes were calculated by dividing the number of cases by the total number of layers in the slaughter batch, including DOA birds. To explore seasonal variations, we generated a variable for season from the slaughter date by dividing the year into four seasons (winter = December to February, spring = March to May, summer = June to August, autumn = September to November). Descriptive statistics were performed both overall and on batch level. Values more than 1.5 IQR above the third quartile were defined as outliers and omitted (two slaughter batches). Additionally, one slaughter batch contained only 273 layers, which was considered unlikely and omitted for that reason (Fig. 1).

To explore a potential effect of season on DOA birds and total carcass condemnation, we used mixed effects negative binomial models performed for each outcome variable (DOA and total carcass condemnation) separately. Negative binomial models were chosen over Poisson models due to over dispersion, i.e., conditional variance of the outcome variable was larger than the conditional mean. Season was included as fixed and farm as a random effect in the models. Potential confounders like hybrid, transportation distance and temperature were not included in the models as such information could not be obtained. Both DOA and total carcass condemnation were counts per slaughter batch, and the number of layers shipped in each batch were defined as exposure in the model (i.e., ln(exposure) is the offset variable). Regression diagnostics were performed for each model by computing conditional Pearson residuals to determine whether the models adequately represented the data. To assess residuals, normality plots were used and plotted against predicted values to evaluate the assumption of homoscedasticity. In negative binomial models, the incidence risk ratio is derived from exponentiation of the coefficients. However, as we in the present study utilized cross-sectional data, we have instead chosen to use the term prevalence ratios to describe the difference in predicted prevalence between seasons. Correlation between mean carcass weight and DOA, as well as correlation between total carcass condemnation (except DOA) and DOA was explored graphically and by calculating Spearman’s rank correlation coefficient (ρ). Correlation between mean carcass weight and the other different condemnation causes was assessed visually with scatter plots, with no indications of association detected (results not shown).

## Results

**Fig. 2:**
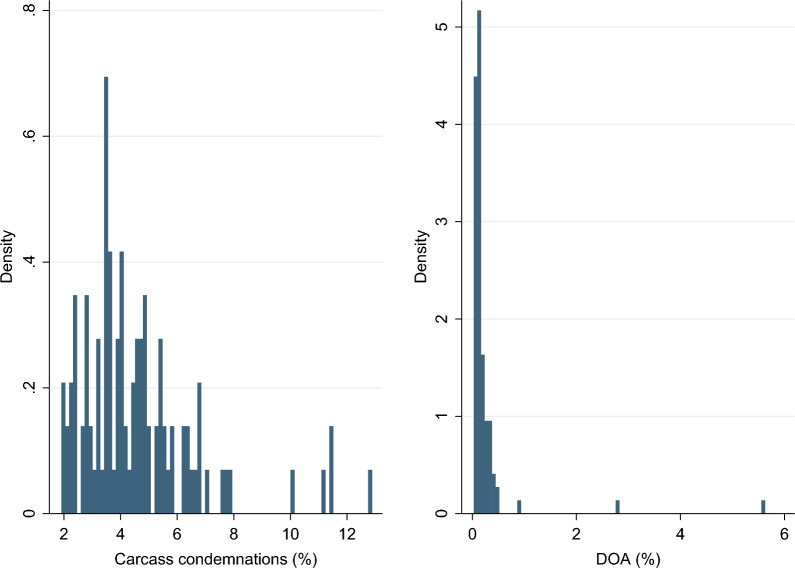
Histogram of carcass condemnation and dead on arrival. Figure describing the frequency distributions of the proportion of total carcass condemnations (left) and the proportion of dead on arrival (DOA) (right). Proportions are on batch level, i.e., the number of cases divided by all layers shipped to the slaughterhouse. The condemnation causes “damage to the carcass” and “fecal contamination” are included in the frequency distribution of the proportion of total carcass condemnations (left)

An overview of the study sample is shown in Table 1. In total, 759,584 commercial layers were delivered to the slaughterhouse in the study period. This constituted 72% of all slaughtered layers in Norway, and approximately 6% of the whole commercial layer population in Norway in that period [4]. The number of layers delivered to slaughter ranged from 273 to 15,514 per slaughter batch. The layers originated from 56 farms, 98 flocks and 101 batches. Three flocks were split and slaughtered in two batches each, explaining the number of batches exceeding the number of flocks. Twenty-four, 40 and 37 batches were slaughtered in 2018, 2019 and 2020, respectively. Possible time trends in carcass condemnations and DOA were visually assessed with box plots, however the distribution of DOA was similar across years (data not shown). The overall mean carcass weight for all slaughtered layers was 977 g, with a variation in batch-mean ranging from 862 to 1047 g. between batches. Of all layers transported to slaughter, 0.22% were DOA. As can be seen in Fig. 2, most batches had a low mortality during transport with a median DOA of 0.13%. However, a few flocks had a notably higher mortality, and DOA ranged from 0.03% to 5.63%. The total number of carcass condemnations was 4.4%. The prevalence of carcass condemnation differed a lot between batches, ranging from 1.91% to 12.86% (Fig. 2). In total, 14 different causes of condemnations were recorded. The causes “damage to the carcass” and “fecal contamination” will not be discussed further as they were deemed of less importance to animal health and welfare since they are damages that occur after slaughter, i.e., damages referring to handling of carcasses and slaughter technique. The overall prevalence of the 12 remaining condemnation causes is shown in Table 2. Descriptive statistics of within-batch prevalence of different carcass condemnation causes is shown in Table 3. The most common cause of condemnation was abscess/cellulitis recorded in 2.03% layers. This was the only condemnation cause present in every slaughter batch.

**Table 1:** Overview of the study sample in a study investigating carcass condemnation in aviary housed layers

	Total number	Mean	Median	Range		St. dev
				Minimum	Maximum	
Farms	56					
Flocks	98					
Slaughter batches	101					
Layers delivered to slaughter	759,584	7520.6	7201	273	15,514	2631.7
Dead on arrival (DOA)	1705	16.9	9	1	396	42.78
DOA % (slaughter batch level)	–	0.24%	0.13%	0.03%	5.63%	0.61%
Layers condemned^a^	33,754	334.2	289	9	970	180.6
Average batch carcass weight (g)	–	977.3	982.8	861.6	1047.3	35.6

^a^Including DOA

**Table 2:** Overall prevalence of condemnation codes from all aviary housed layers (n = 759,584)

Cause of condemnation^a^	Layers	Proportion (%)
Abscess/Cellulitis	15,402	2.028
Peritonitis	2894	0.381
Dead on arrival	1705	0.224
Emaciation	1640	0.216
Discoloration/smell	1600	0.211
Acute skin lesions	1586	0.209
Ascites	1292	0.170
Tumors	323	0.043
Fractures	103	0.014
Salpingitis	75	0.010
Hepatitis	44	0.006
Arthritis	37	0.005

^a^Layers slaughtered in one slaughterhouse in Norway from the period 01.01.2018–31.12.2020. Dead on arrival layers are included in the total number of layers (n = 759,584). One cause of condemnation was recorded per bird

**Table 3:** Descriptive statistics of within-batch prevalence of carcass condemnation causes from all delivered to slaughter (n = 101 batches)

Condemnation	Median (%)	Range		Mean	St. dev	IQR	
		Minimum (%)	Maximum (%)			25 (%)	75(%)
Cellulitis	1.77	0.27	9.07	2.06	1.50	1.02	2.66
Peritonitis	0.35	0.00	1.25	0.39	0.22	0.24	0.47
Discoloration/smell	0.18	0.00	0.94	0.22	0.17	0.11	0.30
Emaciation	0.18	0.00	0.94	0.22	0.16	0.11	0.29
Acute skin lesions	0.17	0.00	0.89	0.20	0.15	0.10	0.25
Circulatory/ascites	0.16	0.00	0.48	0.17	0.11	0.08	0.24
Dead on arrival	0.13	0.03	5.63	0.24	0.61	0.08	0.19
Tumors	0.03	0.00	0.37	0.04	0.06	0.01	0.05
Fractures	0.01	0.00	0.16	0.01	0.02	0.00	0.02
Salpingitis	0.00	0.00	0.08	0.01	0.02	0.00	0.01
Arthritis	0.00	0.00	0.06	0.01	0.01	0.00	0.01
Hepatitis	0.00	0.00	0.06	0.01	0.01	0.00	0.01

**Fig. 3:**
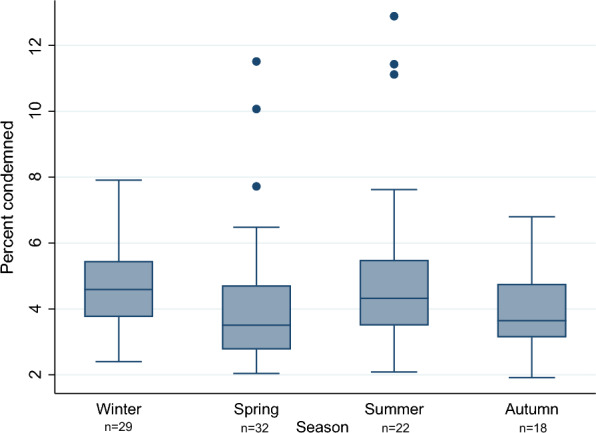
Carcass condemnations in each season. Box plot of the within-batch prevalence of carcass condemnation for each season, in a study investigating carcass condemnation causes in Norwegian commercial layers. Winter, December–February (n = 29); spring, March–May (n = 32); summer, June–August (n = 22) and autumn, September–November (n = 18)

Overall, there were 29 batches slaughtered during winter, 32 batches in the spring, 22 batches in the summer and 18 batches in the autumn. The within-batch prevalence of carcass condemnations in each season is shown in Fig. 3. There were three extreme values for the number of total carcass condemnations in the spring and three extreme values of total carcass condemnations in the summer. We did not have any information in the dataset explaining these outliers, and they originated from different farms and different dates.

**Fig. 4:**
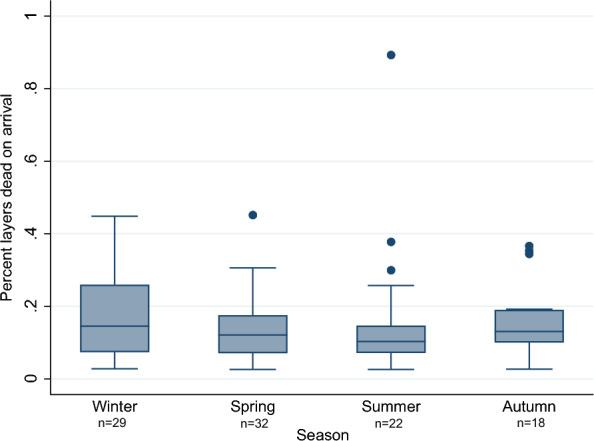
Dead on arrival for each season. Figure showing percentage of layers dead on arrival on batch level for each season, in a study investigating carcass condemnation causes in Norwegian commercial layers. Winter, December–February (n = 29); spring, March–May (n = 32); summer, June–August (n = 22) and autumn, September–November (n = 18). Two outliers (above 95 percentile) in summer were removed

No large differences were detected for the within-batch prevalence of layers recorded as DOA between each season (Fig. 4). Two extreme values of 2.8% and 5.6% DOA recorded during summer, were omitted to ease readability of the figure. These two events originated from different farms but happened on the same date. Their total proportion of carcass condemnation, excluding DOA, were 2.67% and 7.26% respectively.

**Fig. 5:**
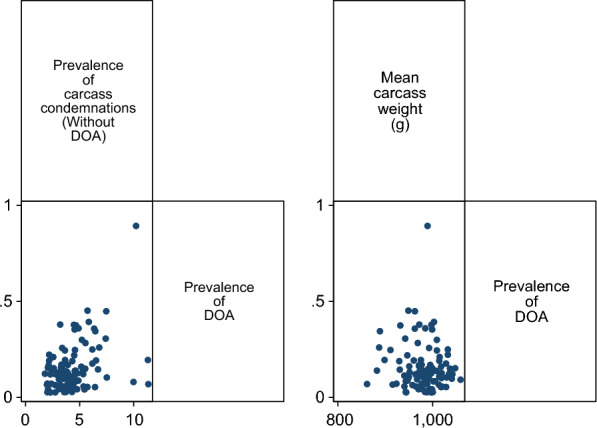
Dead on arrival (DOA) correlation. Matrix graph visualizing the correlation between prevalence of carcass condemnations and DOA (left) (ρ = 0.26) and mean carcass weight and DOA (right) (ρ = − 0.06). DOA birds were removed from the carcass condemnation variable to explore their relationship. Two extreme values of 2.8% and 5.6% DOA were omitted to ease readability of the figure

The correlation between mean carcass weight and DOA was negligible (ρ = − 0.06, P = 0.5769), and a moderate correlation was observed between prevalence of carcass condemnations and DOA (ρ = 0.26, P = 0.0109) (Fig. 5).

**Fig. 6:**
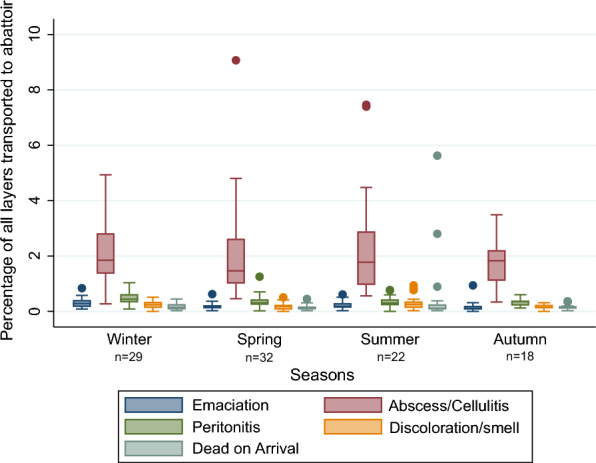
Carcass condemnation causes for each season. Figure showing the most common carcass condemnation causes (% per slaughter batch) for each season, in a study investigating carcass condemnation causes in Norwegian aviary housed layers. Winter, December–February (n = 29); spring, March–May (n = 32); summer, June–August (n = 22) and autumn, September–November (n = 18)

There were no large differences detected between seasons for the distribution of the five most common carcass condemnation causes, on batch level (Fig. 6). Information on hybrids was not available; however, the two hybrids used in commercial egg production in Norway are Lohmann LSL and Dekalb white [4]. The birds were not beak trimmed according to Norwegian legislation [20].

The results from the negative binomial regression models assessing the effect of season on DOA birds and total carcass condemnation are shown in Table 4. In the model assessing the effect of season on DOA birds, spring had the lowest estimated prevalence ratio indicating fewer DOA during spring compared to winter (PR: 0.696, SE: 0.118, 95% CI 0.5–0.97, P: 0.032). The model for total carcass condemnation showed a higher estimated prevalence of condemnations during the winter, compared to the other seasons.

**Table 4:** The effect of season on dead on arrival (DOA) and total carcass condemnation

Outcome	Variables	Prevalence ratio	SE	p-value
DOA	Seasons			
	Winter	Baseline		
	Spring	0.696	0.118	0.032
	Summer	0.873	0.172	0.491
	Autumn	0.847	0.183	0.442
	Random effect variances			
	Farm	0.105	0.069	
Total carcass condemnation	Seasons			
	Winter	Baseline		
	Spring	0.862	0.079	0.103
	Summer	0.971	0.105	0.782
	Autumn	0.792	0.093	0.046
	Random effect variances			
	Farm	0.045	0.021	

Mixed effect negative binomial regression models were used to assess the effect of season on DOA and total carcass condemnation. Both models included farm as random effect. Unit of analysis is based on delivered batch level. Study sample consisted of 98 batches and 53 farms

## Discussion

The objective of the current study was to describe the frequency and causes of carcass condemnation, including DOA, in aviary housed layers in Norway using meat inspection data from a poultry abattoir. The most common cause of carcass condemnation was abscess/cellulitis (2.03%). This was also the only condemnation cause recorded in every batch. Cellulitis in the subcutaneous tissue of broiler chickens is mostly caused by *Escherichia coli*, reaching the subcutaneous tissue through trauma or scratches [21–23]. Little information is available in the literature on prevalence of cellulitis in layers as a condemnation cause. However, cellulitis is a common condemnation cause in broilers [24], where its development is associated with farm management [21, 25]. The condemnation codes are standardized for broiler meat production and are not adapted to the conditions typically found in laying hens. The accuracy of the recording of different condemnation causes could be improved by adapting the codes to laying hens, e.g., having a single condemnation code for abscesses instead of pooling it together with cellulitis. Personal communication with the Norwegian Food Safety Authority at the poultry abattoir revealed that most of the layers in the present study had abscesses in the skin above the sternum, rather than cellulitis. The sternal bursa, acting as a cushion for the sternal crest, can get inflamed after excessive wear from pressure during perching. The perch design and material are associated with plumage damage [26]. Poor plumage condition exposes the skin which in turn can tear and get infected, resulting in abscesses. A study from Denmark reported inflammation of the sternal bursa to be the most common lesion in end-of-lay layers culled on farm [27]. The large between-flock variation in the occurrence of abscess/cellulitis in our sample indicates a potential for prevention on farm level. Saraiva et al. [10] reported cellulitis to be a less common condemnation cause in their sample of layers in cages, barns, free-range and organic free-range systems in Portugal. Their study reported peritonitis as the most common condemnation cause, which was the second most common in the present study (0.38%) [10]. Peritonitis as a cause of on-farm mortality is usually due to infections, appearing together with salpingitis and/or oophoritis where *E. coli* is a common causative agent [12, 13]. As only one cause of condemnation was recorded per bird, it is possible that the layers with peritonitis had comorbidities e.g., salpingitis; however, the more severe lesion was recorded as the cause of condemnation. Salpingitis was recently found to be a common cause of mortality in Norwegian layers, close to slaughter age [16]. Another common condemnation cause in the current study was emaciation (0.22%). Emaciation is often secondary to a primary cause [28, 29]. Reimers et al. [29] reported intussusception of the proventriculus as a cause of emaciation and sporadic mortality. Saraiva et al. [10] also reported emaciation as a common condemnation cause. Emaciation was reported to be a common pathological condition in a study on Norwegian commercial layers found dead at farm end-of-lay [16]. Ascites was also a common condemnation cause in the current study (0.17%). Ascites is a common condemnation cause in broilers [24, 30] and can develop due to metabolic stress resulting in pulmonary hypertension [30]. In layers, ascites has been associated with ovarian and oviduct carcinoma [31], which layers may be susceptible to due to high ovulatory rate [32]. There were no data in our study that either support or contradict this association. Saraiva et al. [10] also reported ascites as a common condemnation cause and found it to be more common in older layers (87–131 weeks).

Layers condemned due to DOA were not necropsied or otherwise examined. Therefore, possible pathological findings in those layers are unknown. DOA layers could have been condemned for other reasons, had they survived the transport, thus, potentially causing the prevalence of different conditions recorded at slaughter (condemnation causes) to differ from the prevalence on farm before transport. A recent study on layers dead on farm at end-of-lay in Norway reported fatty liver, emaciation and salpingitis to be common lesions and salpingitis and fatty liver hemorrhagic syndrome were common causes of death [16]. Another study from Denmark on *post-mortem* findings in culled layers at end-of-lay reported presternal bursitis, infection in the reproductive tract (e.g. salpingitis) and scarification/serosal fibrosis on abdominal organs (e.g. peritonitis) to be the most common pathological findings in layers housed in aviary systems [27]. We found a positive correlation between number of carcass condemnations and DOA, showing that batches with high number of DOA birds also had more carcass condemnations. This could indicate that there are some common risk factors associated with both death during transport and carcass condemnation. Further statistical analysis to shed light on this would be preferable, however it would require information on possible risk factors like management, transportation data and hybrid, which were not available in the current study. As carcass condemnation to some extent reflects the flock health, one could ask if flocks with high DOA-numbers were fit for transport or should have been euthanized on farm. According to legislation, only animals fit for travel can be transported [33]. However, assessing the bird’s fitness for transport can be challenging and the decision relies on a subjective assessment of the flock. The results of the present study showed a mean DOA to be 0.24%, with a substantial variation between batches (range: 0.03–5.63%). This is similar to the results from a study by Weeks et al. [3] who reported an overall mean of 0.27% DOA from a large sample (n = 13.3 million) of layers from caged, intensive indoor, barn, free-range and organic free-range systems in Great Britain. Several studies have identified long travel distance and low external temperatures as two main risk factors for DOA prevalence in layers [3, 6–9]. A study from Italy investigating DOA in broilers, turkeys and layers, reported that mortality from farm to slaughter was higher in summer months compared to other seasons for all investigated birds [34]. This discrepancy to the previously mentioned studies was further discussed as an outcome of heat stress as the temperature and relative humidity is usually high in Italy during summer [34]. In the present study, the highest median prevalence of DOA, and the largest variation in DOA was found during winter, however, the seasonal variations were generally small. In our regression analysis on the effect of seasons on DOA birds, spring had significantly lower DOA numbers compared to winter. However, our sample was quite small and important confounders like hybrid, temperature and transportation time were not included in the model as that information could not be obtained. The same weakness applies to the regression analysis of seasonal effect on total carcass condemnation. In this analysis we found that there were more carcass condemnations during winter compared to the other seasons. However, with the relatively few batches slaughtered each season and lack of information on important confounders, these results should be interpreted as explorative and further studies are needed to fully assess seasonal effects. Effects of other risk factors such as production data (including on farm mortality) and transportation data could not be obtained and was therefore not investigated. All data was aggregated at slaughter batch-level, and not each individual transportation vehicle. We therefore do not know which flocks were transported in more than one vehicle and if there was a difference in DOA numbers between vehicles for the same batch. Information on age at slaughter was also not obtainable, however, layer flocks are typically euthanized or slaughtered at 75–80 weeks of age in Norway [4]. Another recent study reported the overall average mortality from farm to slaughter to be lower than our results (0.17%), however travel distances in their sample were generally short [10]. They also found that body weight had an effect on the risk of DOA, with lighter hens presenting higher risk of mortality [10]. The same was reported in the previously mentioned study by Weeks et al. [3]. We did not find correlation between mean carcass weight and mortality during transport (i.e., DOA). Unfortunately, we were not able to obtain any data on transportation vehicles, -length, or -time. Meteorological data can be obtained for daily air temperature variations in different regions. However, as we did not have information on which time during the day the layers were transported, nor from which region, we decided not to obtain and include such data in our analysis except for the two outliers omitted in Fig. 3. Those two outliers originated from the same date during summer. To check for extreme temperature conditions historical meteorological data from that date was obtained. The air temperature variation was 11.3–22.7 °C in the region where the slaughterhouse is located. Two other batches were delivered on the same date and had a mortality rate at 0.07% and 0.12%, which is within the IQR for that season (0.07–0.23%). Layers at end-of-lay can be susceptible to cold stress during transport due to little fat and bad feather cover [3, 35]. Information on the container’s temperature was not available in the accessed data. Injuries from handling the layers can also contribute to DOA. In contrast to the above-mentioned condemnation causes, acute skin lesions are mainly due to equipment, crating, transportation, or uncrating at slaughterhouse and are indicators of the welfare from farm to slaughter. There was a considerable variation between batches in carcasses condemned due to acute skin lesions. This might indicate that prevention may be possible, and that an assessment and improvement of the handling of the hens during catching, crating and transport could be of benefit. In this regard, catching method might be of particular importance as it is a well-known risk factor for injuries [36, 37]. We did not have information on catching method or handling of the layers in this study, however, all slaughtered laying hens in Norway are caught manually as the catching machines do not fit in the houses.

The most important factor that could compromise the external validity of the present study is the risk of selection bias. The study sample constituted approximately 6% of the Norwegian commercial layer population in that period [4]. Additionally, the data was collected from a single slaughterhouse in one region of the country. Another slaughterhouse might have had differences in inspection practices, as previously shown for broiler production [38–40]. Altogether, these factors imply a cautious approach to generalization of the study results. The number of flocks slaughtered was at any given time driven by the demand for laying hen meat products in the market. Such market drivers will make selection bias due to health on farm less likely, however there is a possibility that an unhealthy flock is euthanized on farm rather than sent to slaughter, e.g., if the majority of the flock is not fit for transport. Nevertheless, this is the first study to report DOA and causes of carcass condemnation in Norwegian aviary housed laying hens based on meat inspection data. The present study provides new insight to important health issues in layers which is information that is scarce in the scientific literature. Furthermore, the high occurrence of abscess/cellulitis in the present study points to a need to investigate the causal mechanisms behind this, including effect of variables related to flock management. Other important areas of further research are to investigate the effect of potential risk factors for carcass condemnation such as hybrid and age to inform targeted preventive measures.

## Conclusions

The most common condemnation causes were (overall proportion): abscess/cellulitis (2.03%), peritonitis (0.38%), DOA (0.22%), emaciation (0.22%), discoloration/smell (0.21%) acute skin lesions (0.21%) and ascites (0.17%). The large between-batch variation in causes of condemnation and DOA indicates a potential for prevention and the results highlight a direction for future studies to further inform flock health and welfare management of layers.

## Data Availability

The dataset used and analyzed during the current study is available from the corresponding author on reasonable request.
